# Pharmacists’ knowledge and counselling on fall risk increasing drugs in a tertiary teaching hospital in Nigeria

**DOI:** 10.1186/s12913-020-05140-6

**Published:** 2020-03-30

**Authors:** Wuraola Akande-Sholabi, Francis S. Ogundipe, Rasaq Adisa

**Affiliations:** Department of Clinical Pharmacy and Pharmacy Administration, Faculty of Pharmacy, University of Ibadan, Ibadana, Nigeria

**Keywords:** Pharmacist, Fall-risk-increasing drugs, Orthostatic drugs, Counselling

## Abstract

**Background:**

Falls and fall-related injuries are a foremost health concern among older adults aged 60 years and above. Fall-risk-increasing drugs (FRIDs) use by older adults is one related cause of falling, and it is frequently used among older adults. Pharmacist-led counselling is an aspect of patient education that has been associated with improved therapeutic outcome and quality of life in high income countries with scarcity of information in low-middle income countries. This study therefore aims to assess hospital pharmacists’ knowledge and counselling on fall-related medications using the list compiled by the Swedish National Board of Health and Welfare on FRIDs and orthostatic drugs (ODs).

**Methods:**

A cross-sectional survey was carried out among 56 pharmacists working in a teaching hospital in Nigeria, between July and August 2019, using a self-administered questionnaire. Data were summarized with descriptive statistics while chi-square test was used for categorical variables at *p* < 0.05.

**Results:**

Thirty-five (62.5%) were within 10 years of practice experience. Two-third (62.5%) of the pharmacists possessed an additional qualification to Bachelor of Pharmacy degree. Twenty-two (40.0%) were aware of the FRIDs and ODs list. In all, (89.3%) had “unsatisfactory” knowledge of classes of medications and specific medicines that could cause a fall. Most pharmacists 42 (80.8%) focused counsel on appropriate medication use, adverse effects of drugs and storage of medications. Knowledge score of both FRIDs and ODs were neither significantly associated with pharmacists’ years of qualification (χ^2^ = 1.282; *p* = 0.733), (χ^2^ = 2.311; *p* = 0.510) nor with possession of additional qualification (χ^2^ = 0.854; *p* = 0.836), (χ^2^ = 2.996; *p* = 0.392). Majority, 53 (98.1%) believed that patients will benefit from effective counselling on FRIDs and ODs. About half (25; 51.0%) suggested training through seminar presentation as a measure for FRIDs and ODs sensitization.

**Conclusion:**

A substantial gap in knowledge and awareness of FRIDs and ODs was noted among the hospital pharmacists. However, engagement of pharmacists on counsel that focus on medication use, adverse effect and storage was relatively better. Thus, there is a general need to create awareness about fall-risk-increasing drugs among hospital pharmacists, so as to help improve the therapeutic outcome particularly in the older adults.

## Background

Pharmacist-led counselling is an aspect of patient education that has been identified to improve drug/disease understanding, clinical outcomes, patients’ quality of life, and decrease health service utilization [1, 2]. The Joint International Pharmaceutical Federation (FIP)/World Health Organization (WHO) guidelines for pharmacy practice has shown that a satisfactory pharmacy practice is pharmacist contributing to health improvement and assisting patients with health challenges to make the best use of their medicines [3].

Medicine-related fall is a preventable multi-factorial cause of substantial morbidity and mortality, especially in older adults aged 60 years and above [4, 5]. Fall is responsible for a considerable ratio of hospital-acquired injuries and the rates of fall are more in hospital in-patients than the community [6–8]. Medications that affect the central nervous system are recognized to enhance the risk of fall, however, pharmacist-led medication improvement programmes have enriched medication use, discontinuation of fall-risk-increasing drugs (FRIDs), as well as medications that can cause orthostatic (ODs) [9]. Fall is a regular adverse drug event observed mostly in the older adults, that might be caused by pharmacogenetic variations impairing drug elimination from some enzymatic modifications or causing some disease and drug-drug interactions [10].

Physicians and patients generally overemphasize the advantages of medications and underrate the potential harms [11]. Moreover, several physicians perceive the uncertainty about the implications of discontinuing FRIDs and ODs as challenging [12]. Subsequently, inadequate knowledge and skills in FRIDs and ODs withdrawal and unwillingness to withdraw implies preventable injurious events and other adverse outcomes related to falls remain [13]. Cardiovascular and psychotropic medicines are the most commonly reported medications in the fall-risk-increasing drugs [13–19]. Fall prevention involves a multidisciplinary approach of healthcare professionals such as pharmacists, physicians, physiotherapists, occupational therapists and nutritionists [14]. Pharmacist interventions in terms of providing advice on appropriate medication changes could reduce falls by up to 70%, while tapering of dose and discontinuation of a medication may also be part of such intervention [20]. Thus, all health professionals involved in the management of patients at risk of falling should develop and maintain the basic skills and competence in fall assessment and prevention. This might have informed the increasing consideration being paid to fall-risk-increasing drugs (FRIDs) and medications that can cause orthostatic (ODs) [13]. However, criteria defining the appropriate or inappropriate use of both FRIDs and ODs in Nigeria are not readily available, while pharmacist’s role in fall prevention programmes are not obvious. Notably, no study has been found on knowledge of FRIDs and ODs among the hospital pharmacists in Nigeria. This study therefore aims to assess the knowledge and counselling on fall-related medications among the pharmacists in a tertiary teaching hospital in Ibadan, Nigeria using the list of drugs compiled by the Swedish National Board of Health and Welfare on FRIDs and ODs. In addition, association between knowledge and counselling on FRIDs and participants’ demographic characteristics was explored.

## Methods

### Study design

This study was a questionnaire-guided cross-sectional survey among pharmacists working in a teaching hospital between July and August 2019.

### Study setting

The University College Hospital (UCH) is a 900-bed premier teaching hospital in Nigeria, affiliated with University of Ibadan. UCH is a federal healthcare institution strategically located in Ibadan, within the south-west geopolitical zone in the country, and it is a major site for undergraduate and post-graduate residency training for physicians, as well as clinical training for other categories of healthcare practitioners including pharmacists, nurses and other auxiliary healthcare personnel.

### Study population

Pharmacists practicing at the University College Hospital, Ibadan.

### Inclusion and exclusion criteria

Eligible participants were registered hospital pharmacists, who gave voluntary informed consent to participate in the study. Participating pharmacists must have had a minimum of one-year practice experience in the hospital pharmacy. Pharmacy students, interns, and hospital pharmacists who were absent from their duty post and those who declined participation were excluded.

### Sample size determination

Number of pharmacists working at the hospital was obtained from the hospital management. Based on the estimated population of 90 registered pharmacists and using the assumption of 95% confidence level and 5% margin of error, a sample size of 73 was obtained using Yamane sample size formula [21]. Adjusting for a 10% non-response rate gave a target sample population of approximately 81. However, out of the sampling frame of 90 pharmacists, six were part of the pre-test and were excluded from the main study, seven pharmacists were on annual leave during the study period, six were on outside posting and five did not consent to the study. Thus, giving a total of 66 eligible participants to guide enrolment.

### Data collection instrument

The main instrument used for data collection was a structured questionnaire developed by the investigators following extensive review of relevant studies [5, 8, 13], as well as employing previous experience. The questionnaire consisted of four sections. Section A captured demographic characteristics, years of experience in hospital practice, and educational qualifications. Section B comprised questions that assessed pharmacists’ awareness, training and perception on FRIDs and ODs. Section C contained questions that assessed pharmacists’ counselling and dispensing of FRIDs and ODs. Section D evaluated general knowledge on the classes of medications and medications that can cause fall and orthostatic hypotension (See Additional file 1).

### Pretest and content validation

The questionnaire was assessed for content validity by two pharmacists in academia chosen from the department of Clinical Pharmacy and Pharmacy Administration, University of Ibadan, to ascertain the comprehensiveness of question-items vis-à-vis the study objectives, as well as ensuring that there are no ambiguous questions or statements. Subsequently, the questionnaire was given to six pharmacists chosen from the University College Hospital, Ibadan to ascertain the ease of comprehension of the item-statements, these pharmacists were not included in the main study. Feedback from the pretest and content validation led to minor modifications including some questions initially designed in open-ended format which were subsequently re-modified as a dichotomous Yes/No format to eliminate response ambiguity.

### Sampling and data collection procedure

Purposive sampling technique/approach was used for participants’ enrolment. Eligible hospital pharmacists were approached by visiting individual pharmacist in their respective practice unit. Objectives of the study were explained to every pharmacist after which voluntary verbal informed consent was obtained to signify intention to participate in the study. The questionnaire was self-administered by all consented pharmacists and retrieved within 25–30 min of completion of the questionnaire. Anonymity and confidentiality of response were assured, while participation was entirely voluntary.

### Data analysis

At the end of each day of the study, the administered questionnaires were sorted, crosschecked after each interview and coded serially. Data entering, cleansing and analysis was done using IBM SPSS (version 23). Descriptive statistics including frequency and percentage were used to summarise the data. In this study, the overall score by pharmacists in the knowledge domains developed for the purpose of this study was converted into percentage to ensure uniformity in the scores. In the knowledge domain, a total score ≥ 80% was considered as “satisfactory” knowledge, while score < 80% signified “unsatisfactory” knowledge. Thus, a percent score > 80% indicates a raw score of > 7 out of the 9 questions that evaluated the general knowledge on classes of medications and medications that can cause falls; > 4 out of 5 questions on knowledge on classes of medications and medications that can cause orthostatic. The cut-off criteria for the binary categorization was adapted from Bloom’s cut-off point criteria, as well as review of other related studies [22–24].

Chi-square (χ^2^) test was used to assess association between categorical variables such as demographic characteristics, as well as years of practice, pharmacists’ rank, pharmacists’ awareness on FRIDs and ODs and pharmacists’ previous training on FRIDs and ODs. Level of significance was set at *p* < 0.05.

## Results

### Demographics characteristics of pharmacists

Of the 66 copies of questionnaire administered to the pharmacists, 56 were returned and completely filled, given a response rate of 84.8%. Thirty-eight (67.9%) were females. The mean age was 38.7 ± 6.7 years (range 20–59 years), with majority of the pharmacists (55; 98.2%) aged 30 years and above. Most of the pharmacists (52; 92.9%) were married, while, 35 (62.5%) had additional qualifications to B.Pharm degree. The professional cadre of the pharmacists who participated in the study included principal pharmacists (18; 32.1%), grade 1 pharmacist (13; 23.2%), chief pharmacist (12; 21.4%), assistant deputy director (7; 12.5%) and deputy director (6; 10.7%). A total of 35 (62.5%) had practice experience over 10 years post intern’s qualification, while 17 (30.4%) had practice in UCH for over 10 years. Out of the 18 satellite pharmacy units explored 16 (28.6%) pharmacists worked at the General outpatient department, 7 (12.5%) worked at the cash and carry pharmacy. Details of demographic characteristics are shown in Table 1.

**Table 1:** Demographic characteristics of pharmacists (*n* = 56)

Variables	Category	Frequency (%)
Gender	Male	18 (32.1)
	Female	38 (67.9)
Age (year)	20–29	1 (1.8)
	30–39	35 (62.5)
	40–49	16 (28.6)
	50–59	4 (7.1)
Marital status	Single	3 (5.4)
	Married	52 (92.9)
	Widowed	1 (1.8)
Educational Level	B. Pharm	21 (37.5)
	Pharm D	5 (8.9)
	Masters	22 (39.3)
	WAPCP	8 (14.3)
Years of practice post Induction	1–5	4 (7.1)
	6–10	17 (30.4)
	11–15	20 (35.7)
	> 15	15 (26.8)
Pharmacists’ rank	Grade 1 Pharmacist (0-3 years)	13 (23.2)
	Principal Pharmacist (3-6 years)	18 (32.1)
	Chief Pharmacist (6-9 years)	12 (21.4)
	Asst. deputy director (9-12 years)	7 (12.5)
	Deputy director (12-15 years)	6 (10.7)
Years of Practice in UCH	< 1	6 (10.7)
	1–5	21 (37.5)
	6–10	12 (21.4)
	11–15	7 (12.5)
	> 15	10 (17.9)
Satellite pharmacy units	General Outpatient	16 (28.6)
	Cash and carry	7 (12.5)
	Medical Outpatient	6 (10.7)
	Neurosurgery Inpatient	4 (7.1)
	Geriatric	3 (5.4)
	Private suite	3 (5.4)
	Pediatrics	2 (3.6)
	Staff clinic	2 (3.6)
	Medical Inpatient	2 (3.6)
	Accident and Emergency	2 (3.6)
	Compounding/manufacturing	2 (3.6)
	Surgical Inpatient	1 (1.8)
	Surgical Outpatient	1 (1.8)
	Informatics	1 (1.8)
	Oncology	1 (1.8)
	Ear, Nose, Throat and Eye	1 (1.8)
	Dental	1 (1.8)
	Critical care & surgeries	1 (1.8)

^a^*WAPCP=* West African Postgraduate College of Pharmacist, *UCH=* University College Hospital

### Awareness, training and perception of fall risk increasing drugs (FRIDs) and orthostatic drugs (ODs) among the pharmacists

Twenty-two pharmacists (40.0%) were aware of FRIDs and ODs list, of this, most (6; 28.5%) became aware through drug leaflet. Although, 50 (90.9%) and 47 (90.4%) of respondents claimed to be aware of medications that can cause falls and orthostatic hypotension, respectively, only 5 (9.3%) had previous training on FRIDs and ODs. Majority (53; 98.1%) of the pharmacists believed that patients will benefit from effective counselling on FRIDs and ODs (Table 2).

**Table 2:** Awareness, training and perception of fall-risk-increasing drugs and orthostatic drugs among the pharmacists

Variables	Response	Frequency (%)
Awareness of the list of FRIDs and ODs (*n* = 55)	Yes	22 (40.0)
	No	33 (60.0)
Sources of awareness of the list of FRIDs and ODs (*n* = 21)	Drug leaflets	6 (28.5)
	Journal articles	5 (23.8)
	Textbooks	5 (23.8)
	Drug company presentations	3 (14.3)
	Colleagues	1 (4.8)
	Class lecture	1 (4.8)
Previous training on FRIDs and ODs (*n* = 54)	Yes	5 (9.3)
	No	49 (90.7)
Place of previous training on FRIDs and ODs (*n* = 5)	Pharmacy school	3 (60.0)
	Seminars	1 (20.0)
	Pharm D program	1 (20.0)
Awareness of medications causing fall (*n* = 55)	Yes	50 (90.9)
	No	5 (9.1)
Awareness of medications causing orthostatic hypotension (*n* = 52)	Yes	47 (90.4)
	No	5 (9.6)
Pharmacists’ perception of patients benefiting from counselling on FRIDs and ODs (*n* = 54)	Yes	53 (98.1)
	No	1 (1.9)
Suggested measures for FRIDs and ODs sensitization (*n* = 49)	Training through seminar presentation	25 (51.0)
	Continuous professional development	9 (18.4)
	List and colour code drugs in dispensary	8 (16.3)
	Bulletin provision	4 (8.2)
	Social media awareness	2 (4.1)
	Access to drug information services	1 (2.0)

*FRIDs* Fall-risk-increasing drugs, *ODs* Orthostatic drugs, *n* Number

### Extent of counselling and dispensing of fall risk-increasing drugs (FRIDs) and orthostatic drugs (ODs) among the pharmacists

In each pharmacy unit, between 50 to 100 patients attended the unit per day, of this, at least 1–50 patients (38; 67.9%) were estimated to be aged > 60 years among those counselled daily. Majority of pharmacists (48; 85.7%) counselled patients directly in their units, and most counselling took between 1 and 5 min (34; 69.4%). The major challenge encountered during counselling was communication barrier and patient’s impatience (12; 50.0%), followed by lack of time and workload (7; 29.2%), and inconvenient work environment (5; 20.8%). Majority of the pharmacists (42; 80.8%) focused counsel on appropriate medication use, adverse effects of drugs and storage of medications, while (8; 15.4%) counselled on drug interactions and only one (1.9%) pharmacist each counselled on lifestyle modifications as well as discharged counselling. Elderly patients are mostly counselled on common drug-specific adverse effects (9; 32.1%) especially gastro-intestinal discomfort (6; 21.4%). Details are shown in Table 3.

**Table 3:** Extent of counseling and dispensing of FRIDs and ODs among the pharmacists

Variables	Response category	Frequency (%)
Average number of patients attended to daily (*n* = 54)	0	3 (5.6)
	1–50	18 (33.3)
	51–100	21 (38.9)
	101–150	6 (11.1)
	151–200	1 (1.9)
	> 200	5 (9.3)
Patients above 60 years counselled daily (*n* = 55)	0	15 (27.3)
	1–50	38 (67.9)
	51–100	2 (3.6)
Frequency of dispensing FRIDs and ODs (*n* = 49)	Daily	32 (65.3)
	Once in a while	14 (28.6)
	Not at all	2 (4.1)
	Frequently	1 (2.0)
Counseling focus of pharmacists (*n* = 52)	Appropriate medication use, adverse effects and medication storage	42 (80.8)
	Drug interactions	8 (15.4)
	Lifestyle modifications	1 (1.9)
	Discharge counseling	1 (1.9)
Common drug-specific adverse effects encountered by the elderly (*n* = 28)	Common adverse effects of medications	9 (32.1)
	Gastro-intestinal discomfort	6 (21.4)
	Sedation and dizziness	5 (17.9)
	Hypoglycemia and fall	4 (14.3)
	Benzodiazepine dependence	2 (7.1)
	Medication spacing	1 (3.6)
	Constipation	1 (3.6)
Pharmacists counselling patients (*n* = 56)	Yes	48 (85.7)
	No	8 (14.3)
Average duration of counseling (*n* = 48) (minutes)	1–5	34 (70.8)
	6–10	12 (25.0)
	11–15	1 (2.1)
	16–20	1 (2.1)
Time sufficiency during counseling (*n* = 52)	Yes	29 (55.8)
	No	23 (44.2)
Counseling limitations (*n* = 24)	Communication barrier and Impatient patients	12 (50.0)
	Lack of time and workload	7 (29.2)
	Inconvenient work environment	5 (20.8)

### Knowledge assessment of pharmacists on FRIDs

Table 4 shows the knowledge of pharmacists on FRIDs. Of the classes of medications, 43 (76.8%); 27 (48.2%); 20 (35.7%) and 15 (26.8%) had the knowledge that antihypertensives, antipsychotics, antidiabetics and anticholinergics, respectively could cause/predispose to fall. In all, 6 (10.7%) had score ≥ 80% indicating “satisfactory” knowledge of classes of medications and specific medicines that could cause a fall.

**Table 4:** Knowledge assessments on the classes of medications and medications that can cause fall

Class of medications (***n*** = 56)	Response category	Frequency (%)
Antihypertensive can cause fall Correct answer: Yes	Yes	43 (76.8)
	No	13 (23.2)
Antidiabetics can cause fall Correct answer: Yes	Yes	20 (35.7)
	No	36 (64.3)
Anticholinergics can cause fall Correct answer: Yes	Yes	15 (26.8)
	No	41 (73.2)
Antipsychotics can cause fall Correct answer: Yes	Yes	27 (48.2)
	No	29 (51.8)
**Specific medications that can cause fall (*n*** **= 56)**		
Nifedipine Correct answer: Yes	Yes	27 (51.8)
	No	29 (48.2)
Dihydrocodeine Correct answer: Yes	Yes	12 (21.4)
	No	44 (78.6)
Propranolol Correct answer: Yes	Yes	16 (28.6)
	No	40 (71.4)
Glimepiride Correct answer: Yes	Yes	12 (21.4)
	No	44 (78.6)
Bromazepam Correct answer: Yes	Yes	18 (32.1)
	No	38 (67.9)
**Score distribution** **FRIDs Scores (*n*** **= 56)**	**Frequency**	**%**
0	6	10.7
1	2	3.6
2	13	23.2
3	11	19.6
4	6	10.7
5	9	16.1
6	3	5.4
7	3	5.4
8	2	3.6
9	1	1.8
**Knowledge on FRIDs**		
Satisfactory knowledge (score ≥ 80%)	6	10.7
Unsatisfactory knowledge (score < 80%)	50	89.3

Maximum Score for FRIDs = 9, *Individual score* Individual score÷maximum obtainable score × 100 *FRIDs* Fall-risk-increasing drugs

### Knowledge assessment of pharmacists on ODs

Table 5 shows the pharmacists’ knowledge on medications with increasing risk of ODs, with 46 (82.1%) and 10 (17.9%) who had the knowledge that antihypertensive and antipsychotic classes of medications, respectively, could cause orthostatic hypotension. Majority, 50 (89.3%) had score < 80% indicating “unsatisfactory” knowledge of classes of medications and specific medicines that could cause orthostatic hypotension.

**Table 5:** Knowledge assessments on the classes of medications and medications that can cause orthostatic hypotension

Class of medications (***n*** = 56)	Response category	Frequency (%)
Antihypertensive can cause orthostatic hypotension Correct answer: Yes	Yes	46 (82.1)
	No	10 (17.9)
Antipsychotics can cause orthostatic hypotension Correct answer: Yes	Yes	10 (17.9)
	No	46 (82.1)
**Some medications that can cause orthostatic (*n*** **= 56)**		
Prazosin can cause orthostatic hypotension Correct answer: Yes	Yes	28 (50.0)
	No	28 (50.0)
Chlorpromazine can cause orthostatic hypotension Correct answer: Yes	Yes	5 (8.9)
	No	51 (91.1)
Hydralazine can cause orthostatic hypotension Correct answer: Yes	Yes	22 (39.3)
	No	34 (60.7)
**Score distribution** **ODs Scores (*n*** **= 56)**	**Frequency**	**%**
0	8	14.3
1	9	16.1
2	18	32.1
3	15	26.8
4	5	8.9
5	1	1.8
**Knowledge on ODs**		
Satisfactory knowledge (score ≥ 80%)	6	10.7
Unsatisfactory knowledge (score < 80%)	50	89.3

Maximum Score for ODs = 5, Individual score = individual score÷maximum obtainable score × 100

### Factors associated with knowledge of FRIDs and ODs

Gender (χ^2^ = 0.982 *p* = 0.374), educational level (χ^2^ = 0.854 *p* = 0.836), years of practice (χ^2^ = 1.282 *p* = 0.733), and awareness of FRIDs list (χ^2^ = 0.281 *p* = 0.6748) did not significantly influence participants’ knowledge of FRIDs (Table 6). However, participants’ awareness of ODs list significantly affects knowledge of ODs (χ^2^ = 6.615 *p* = 0.003).

**Table 6:** Association between relevant demographic characteristics and pharmacists’ knowledge on fall risk increasing drugs and orthostatic drugs

Variables	Knowledge on FRIDs, n (%)		Knowledge on ODs, n (%)	
	Satisfactory (score ≥ 80%)	Unsatisfactory (score < 80%)	Satisfactory (score ≥ 80%)	Unsatisfactory (score < 80%)
**Gender**				
Male	3 (16.7)	15 (83.3)	0 (0.0)	18 (100.0)
Female	3 (7.9)	35 (92.1)	6 (15.8)	32 (84.2)
	[χ^2^ = 0.982; *p* = 0.374]		[χ^2^ = 3.183; *p* = 0.162]	
**Educational Level**				
B.Pharm	2 (9.5)	19 (90.5)	1 (4.8)	20 (95.2)
Pharm D	0 (0.0)	5 (100.0)	1 (20.0)	4 (80.0)
Masters	3 (13.6)	19 (86.4)	2 (9.1)	20 (90.9)
WAPCP	1 (12.5)	7 (87.5)	2 (25.0)	6 (75.0)
	[χ^2^ = 0.854; *p* = 0.836]		[χ^2^ = 2.996; *p* = 0.392]	
**Years of Practice**				
1-5 years	0 (0.0)	4 (100.0)	0 (0.0)	4 (100.0)
6-10 years	1 (6.3)	15 (93.8)	3 (18.8)	13 (81.3)
10-15 years	3 (15.0)	17 (85.0)	1 (5.0)	19 (95.0)
> 15 years	2 (13.3)	13 (86.7)	2 (13.3)	13 (86.7)
	[χ^2^ = 1.282; *p* = 0.733]		[χ^2^ = 2.311; *p* = 0.510]	
**Pharm Rank**				
Grade 1 Pharm.	1 (7.7)	12 (92.3)	2 (15.4)	11 (84.6)
Principal Pharm	2 (11.1)	16 (88.9)	3 (16.7)	15 (83.3)
Chief Pharm	2 (16.7)	10 (83.3)	0 (0.0)	12 (100.0)
Asst. Director	0 (0.0)	7 (100.0)	1 (14.3)	6 (85.7)
Deputy Director	1 (16.7)	5 (83.3)	0 (0.0)	6 (100.0)
	[χ^2^ = 1.634^;^*p* = 0.803]		[χ^2^ = 3.216; *p* = 0.522]	
**Awareness on FRIDs and ODs**				
Yes	3 (13.6)	19 (86.4)	6 (27.3)	16 (72.7)
No	3 (9.1)	30 (90.9)	0 (0.0)	33 (100.0)
	[χ^2^ = 0.281; *p* = 0.674]		[χ^2^ = 10.102 *p* = 0.003*]	
**Previous training on FRIDs and ODs**				
Yes	1 (20.0)	4 (80.0)	2 (40.0)	3 (60.0)
No	5 (10.2)	44 (89.8)	4 (8.2)	45 (91.8)
	[χ^2^ = 0.844; *p* = 0.385]		[χ^2^ = 6.615; *p* = 0.057]	

*significant difference with Fisher’s Exact *p*-value < 0.05, χ^2^ = Chi square test

## Discussion

Pharmacists are essential member of the healthcare system and are integral in assessing and preventing falls in older adults, considering the role they play in the reduction of polypharmacy, medication therapy management, recommending suitable medication alternatives and counselling on adverse drug reactions [25]. Medication and polypharmacy are the utmost adjustable risk factors for falls and fall-related injuries [26]. The usage of more than one medication or administration of more medications than clinically required is routine in older adults [27]. The usual mechanisms through which medications contribute to falls risk are sedation, hypotension, bradycardia, tachycardia or episodes of asystole [3, 8, 25]. Notably to our knowledge, this is the first study in Nigeria assessing the knowledge and counselling of hospital pharmacists on fall-risk-increasing drugs (FRIDs).

In our study, more than three-quarters of the pharmacists had “unsatisfactory” knowledge of FRIDs and ODs with less than half who were aware of these class of drugs. The low level of awareness and knowledge deficit perhaps signifies the need for creating awareness as well as encouraging consistent training among pharmacists on aspect related to vulnerable population like geriatrics where fall-related injuries are common and can lead to functional decline and reduced quality of life. Although majority of the participants claimed to be aware of the medications that can cause fall and orthostatic hypotension, this does not seem to reflect on their general knowledge on FRIDs and ODs as assessed in our study. It is also noted that nearly one-tenth of the pharmacists had previous training in fall-related medications which may further underscore the need for pharmacist’s engagement in value added service that will enhance patient care. Noteworthy to mention that approximately 98% of the pharmacists believed that effective counselling on FRIDs and ODs will be beneficial for the patient. A finding that is consistent with the report by Laing et al. [28] that less than one-half (38%) of senior serving professionals feel “very knowledgeable” about fall prevention.

Pharmacist trained specifically in pharmacotherapy and medication management can play an important role in fall prevention, through successful medication therapy management of fall-related medicines [25]. Cooper et al. [29] showed that the involvement of pharmacists in the healthcare team to assess fall risks through identifying various predisposing factors and intervening in the medication management helped to reduce falls.

Previous studies have reported cardiovascular and psychotropic medicines as the most common medications in the FRIDs [13–19]. Although more than three-quarters of the pharmacists in our study knew antihypertensives could cause a fall and orthostatic hypotension, however most could not specifically relate other drugs such as antipsychotics, anticholinergics and antidiabetics as risk factors for fall and orthostatic hypotension. Measures suggested by participants for FRIDs and ODs sensitization include training through seminar presentation, continuous professional development, list and color code FRIDs and ODs in the dispensary and inclusion of fall-related medication list in pharmacist bulletin or newsletter. All these measures could improve the awareness of fall-risk-increasing drugs among the pharmacists, with better understanding of the medication and possibly increased knowledge about these medications.

Counselling may perhaps stimulate patients’ interest to adhere to their medications, while updates about potential drug-drug and drug-food interactions will be emphasized [30]. Patient counselling is an important component of pharmaceutical care [31], which mostly create awareness about therapy as well as help the patient to appreciate the purpose of prescribed regimen [32, 33]. Though the significance of counselling practice as a vital component of patient education cannot be overemphasized, the findings in our study showed that counselling was carried out by most of the pharmacists. However, there were some counselling challenges mentioned, which included; excessive workload, communication barrier and patients’ being impatient. Despite these challenges, pharmacists were still able to focus counselling on appropriate medication use, specific drug adverse effects and storage of medications. Previous studies have shown that clinical pharmacist intervention through counselling helped to reduce drug related problems with better clinical outcomes and reduced incidence of falls [34–37]. It has also been documented that pharmacists have effectively reduced, switched and removed fall-related medications, provided clinically imperative recommendations as well as educating patients about medications and the risk of falls [35, 38, 39].

Data from this study shows no statistically significant difference in knowledge of FRIDs and ODs among hospital pharmacists irrespective of their years of practice, experience rank cadre and additional qualifications. This might explain the need for all pharmacists to undergo training on fall-related medications regardless of their years of practice, experience rank or qualifications. Morbidity and mortality associated with falls are increasing worldwide [28]. To curtail the wave of this growth in prevalence, services should be offered to the patients which will focus on effective management strategies aimed at reducing attendant risk associated to fall. Pharmacist, in collaboration with other health care providers, can conduct a medication review, evaluate the patient health conditions, articulate the patient’s medication action plan, and educate the patient about medication changes and fall prevention strategies [25].

### Limitations

This study was conducted among hospital pharmacists in a tertiary teaching hospital perhaps if conducted in more tertiary hospitals, we might have a more comprehensive data of the knowledge of hospital pharmacists in FRIDs and ODs. In addition, other limitations of our study include the smaller sample size but a relatively good response rate, and the possibility of response bias from participants due to over- or under-reporting of the information provided, while the absence of rigorous validation checks for the study instrument aside the content validity and pretest may indicate the need for caution in generalization of the findings to the entire hospital pharmacists in Nigeria. Despite this, the study still offers a key insight into the hospital pharmacists’ knowledge and counselling on fall-risk-increasing drugs, thereby illuminating the area of focus to bridge the knowledge and practice gaps.

## Conclusions

A substantial gap in knowledge and awareness of the FRIDs and ODs was noted among the hospital pharmacists. However, engagement of pharmacists on counsel that focus on medication use, adverse effect and storage was relatively better. Thus, there is a general need to create awareness about fall-risk-increasing drugs among hospital pharmacists, so as to help improve the therapeutic outcome particularly in the older adults.

## Supplementary information

**Additional file 1.** Questionnaire for the participants.

## Data Availability

The datasets used and/or analysed during the current study are available from the corresponding author on reasonable request.

## Abbreviations

FIP: The joint International Pharmaceutical Federation
FRIDs: Fall-Risk-Increasing-Drugs
ODs: Orthostatic Drugs
UCH: University College Hospital
WAPCP: West African Postgraduate College of Pharmacist
WHO: World Health Organization
