# Supplementary material for: Pharmacists’ knowledge and counselling on fall risk increasing drugs in a tertiary teaching hospital in Nigeria
Source: BMC Health Serv Res. 2020 Mar 30;20:259. doi: 10.1186/s12913-020-05140-6 (PMC7106790; doi:10.1186/s12913-020-05140-6)
Supplement: Supplementary file 1 — Additional file 1. Questionnaire for the participants. [file 12913_2020_5140_MOESM1_ESM.docx]

**DEAR RESPONDENTS,**

**This is a questionnaire to assess the knowledge and perception of pharmacists on Fall Risks Increasing Drugs (FRIDs) and Orthostatic Drugs (ODs). This is in partial fulfillment of M. Sc. Programme at the Department of Clinical Pharmacy and Pharmacy Administration, Faculty of Pharmacy, University of Ibadan.**

**Kindly and sincerely help to tick as appropriate/fill in the necessary spaces in each section. Your response will remain anonymous and treated with utmost confidentiality.**

**SECTION A: EVALUATION OF SOCIO-DEMOGRAPHIC INFORMATION**

1. Age: 20-29 yrs ( ) 30-39 yrs (. ) 40-49yrs ( ) 50-59 yrs ( ) 60 yrs and above ()
2. Gender: Male ( ) Female ( )
3. Marital status: Single ( ) Married ( ) Divorced ( ) Widowed ( )
4. Educational level: B Pharm ( ) Pharm D ( ) Postgraduate degrees ( ), Kindly specify for Postgraduate degree ________________________
5. Number of years in practice post induction: Less than a year ( ) 1-5 yrs ( ) 6-10 yrs ( ) 10 -15yrs ( ) more than 15 yrs ( )
6. Pharmacist rank: NYSC Pharmacist ( ) Intern ( ) Pharmacist grade 1 ( ) Principal pharmacist ( ) Chief pharmacist ( ) Assistant director ( ) Deputy director ( ) Director ( )
7. Years of practice in the hospital (University College Hospital): Less than a year ( ) 1-5 yrs ( ) 6-10 yrs ( ) 10-15 yrs ( ) More than 15 yrs ( )
8. Which unit are you currently practicing in the hospital: General Outpatient Department ( ) Medical Outpatient Department ( ) Geriatric Pharmacy ( ) Others, specify ________________________________________________________________________________

**SECTION B**: **ASSESSING PHARMACISTS’ AWARENESS, TRAINING AND PERCEPTION ON FRIDs and ODs**

1. Are you aware that there is a list of FRIDs and ODs? Yes ( ) No ( )
2. If yes, where did you get the information about the list on FRIDs and ODs? Textbooks ( ) Journals ( ) Drug company presentation ( ) Drug leaflets ( ) Others, Specify ____________________________
3. Have you had any previous training on counseling of FRIDs or ODs? Yes ( ) No ( )
4. If yes, where did you receive the training? Undergraduate pharmacy school ( ) Postgraduate pharmacy school ( ) Seminars ( ) Others, Specify _________________________________________________
5. Are you aware of the tendency of some medications to cause falls? Yes ( ) No ( )
6. Are you aware of those medications that cause orthostatic hypotension? Yes ( ) No ( )
7. Do you think patients will benefit more from counseling on the risk of drugs that can predispose them to falls? Yes ( ) No ( )
8. If yes, how can pharmacists be sensitized more on the list of these drugs that predisposes patients to falls?

**SECTION C: ASSESSING PHARMACISTS’ COUNSELLING AND DISPENSING OF FRIDs and ODs**

1. How many patients do you attend to averagely in your current unit? None ( ) 1-50 ( ) 51-100 ( ) 101-150 ( ) 151-200 ( ) More than 200 ( )
2. Do you personally have direct interactive counseling with patients in your unit? Yes ( ) No ( )
3. If yes, how many patients do you counsel averagely in a day in your unit? 1-50 ( ) 51-100( ) 101-150 ( ) 151-200 ( ) More than 200 ( )
4. How many patients above the age of 60 years do you counsel daily on the average in your unit? None () 1-50 ( ) 51-100 ( ) 101-150 ( ) 151-200 ( ) More than 200 ( )
5. What is the average duration used in counseling patients in your current unit? 1-5mins ( ) 6-10mins ( ) 11-15mins( ) 16-20mins ( ) greater than 20mins ( )
6. What is the focus of your counsel? Possible adverse effect of medications ( ) Appropriate medication use( ) Appropriate storage of medications ( ) Others, Specify _________________________________________________________________________________
7. How often do you dispense such drugs that causes fall in your current unit? Not at all ( ) Daily ( ) Once in a while ( ), Frequently ( ) Others, specify ______________________________
8. Do you have sufficient time with your patients during counseling? Yes ( ) No ( )
9. If No, what factor(s) is (are) responsible for the time limitation?

I.

II.

III.

IV.

V.

26. What are the common drug-specific adverse effects encountered by the elderly?

**SECTION D: EVALUATION OF PHARMACISTS’ KNOWLEDGE ON THE CLASS OF MEDICATIONS THAT CAN CAUSE FALL AND ORTHOSTATIC HYPOTENSION.**

1. Which of these classes of medications can cause fall? Antidiabetics: Yes ( ) No ( ) Antihypertensives: Yes ( ) No ( ) Anticholinergics: Yes ( ) No ( ) Antipsychotics Yes ( ) No ( ) Others, Specify ________________________________________________________________________________
2. Which of the following class of drugs can cause orthostatic hypotension? Antihypertensives: Yes ( )No ( ) Antipsychotics: Yes ( ) No ( ) Others, Specify ____________________________________________________________________
3. Which of these medications will most likely cause fall? Nifedipine: Yes ( ) No ( ) Dihydrocodeine: Yes ( ) No ( ) Propranolol: Yes ( ) No ( ) Glimepiride: Yes ( ) No ( ) Bromazepam: Yes ( ) No ( )
4. Which of these medications has been shown to cause orthostatic hypotension?

Prazosin: Yes ( ) No ( ) Chlorpromazine: Yes ( ) No ( ) Hydralazine: Yes ( ) No ( )

**Thank you for your time and response**
